# Influence of metabolic network structure and function on enzyme evolution

**DOI:** 10.1186/gb-2006-7-5-r39

**Published:** 2006-05-09

**Authors:** Dennis Vitkup, Peter Kharchenko, Andreas Wagner

**Affiliations:** 1 Center for Computational Biology and Bioinformatics, Department of Biomedical Informatics, Columbia University, Russ Berrie Pavilion, St Nicholas Avenue, New York, NY 10032, USA; 2 Department of Genetics, New Research Building, Ave Louis Pasteur, Harvard Medical School, Boston, MA 02115, USA; 3 Department of Biology, Castetter Hall, University of New Mexico, Albuquerque, NM 87131, USA

## Abstract

**Background:**

Most studies of molecular evolution are focused on individual genes and proteins. However, understanding the design principles and evolutionary properties of molecular networks requires a system-wide perspective. In the present work we connect molecular evolution on the gene level with system properties of a cellular metabolic network. In contrast to protein interaction networks, where several previous studies investigated the molecular evolution of proteins, metabolic networks have a relatively well-defined global function. The ability to consider fluxes in a metabolic network allows us to relate the functional role of each enzyme in a network to its rate of evolution.

**Results:**

Our results, based on the yeast metabolic network, demonstrate that important evolutionary processes, such as the fixation of single nucleotide mutations, gene duplications, and gene deletions, are influenced by the structure and function of the network. Specifically, central and highly connected enzymes evolve more slowly than less connected enzymes. Also, enzymes carrying high metabolic fluxes under natural biological conditions experience higher evolutionary constraints. Genes encoding enzymes with high connectivity and high metabolic flux have higher chances to retain duplicates in evolution. In contrast to protein interaction networks, highly connected enzymes are no more likely to be essential compared to less connected enzymes.

**Conclusion:**

The presented analysis of evolutionary constraints, gene duplication, and essentiality demonstrates that the structure and function of a metabolic network shapes the evolution of its enzymes. Our results underscore the need for systems-based approaches in studies of molecular evolution.

## Abstract 2

An analysis of evolutionary constraints, gene duplication and essentiability in the yeast metabolic network demonstrates that the structure and function of a metabolic network shapes the evolution of its enzymes.

## Background

Molecular networks and the genes encoding their building blocks represent two different levels of biological organization that interact in evolution. On the one hand, genetic changes such as point mutations, gene deletions, and gene duplications influence the structure and evolution of these networks. Conversely, network function may constrain the kinds of mutations that can be tolerated, and thus how genes evolve. Existing work on the structure and evolution of molecular networks has mainly focused on protein interaction networks [1-6]. Such networks are very heterogeneous: they contain large macromolecular complexes, regulatory interactions, signaling interactions, and interactions of proteins that provide structural support for a cell. As a result, it is difficult to ascertain how network structure reflects network function. A large fraction of false positives and false negatives in protein interaction networks [7,8] further complicates the structure to function analysis. In contrast, cellular metabolic networks are relatively well-characterized in several model organisms such as *Saccharomyces cerevisiae *[9,10] and *Escherichia coli *[11]. Their function - biosynthesis and energy production - is also well understood, as is the relationship of network structure to network function.

In the present study, we ask how the topology of a metabolic network and the metabolic fluxes (a metabolic flux is the rate at which a chemical reaction converts reactants into products) through reactions in the network influence the evolution of metabolic network genes through point mutations and gene duplication. Our results suggest that both network structure and function need to be understood to fully appreciate how metabolic networks constrain the evolution of their parts. The present study has become possible with the recent publication of a comprehensive compendium of metabolic reactions in the yeast *Saccharomyces cerevisiae *[10]. This compendium comprises 1,175 metabolic reactions and 584 metabolites, and involves about 16% of all yeast genes.

Using the stoichiometric equations that describe chemical reactions, we calculate the connectivity of an enzyme as the number of other metabolic enzymes that produce or consume the enzyme's products or reactants (see Materials and methods and Additional data file 1). In other words, a metabolic enzyme A and a metabolic enzyme B are connected if they share the same metabolite as either a product or reactant. Highly connected enzymes in this representation are enzymes that share metabolites with many other enzymes. Including the most highly connected metabolites and cofactors such as ATP or hydrogen in a network representation would render the network structure dominated by these few nodes, and would obscure functional relationships between enzymes. We thus excluded the top 14 most highly connected metabolites: ATP, H, ADP, pyrophosphate, orthophosphate, CO_2_, NAD, glutamate, NADP, NADH, NADPH, AMP, NH_3_, and CoA [12]. The results we report below are qualitatively insensitive to the exact number of removed metabolites.

## Results

### Highly connected enzymes evolve slowly

**Figure 1:**
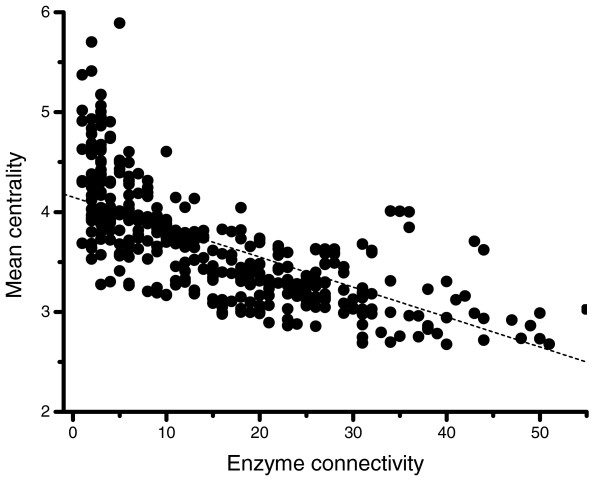
The correlation between enzyme connectivity and centrality in the yeast metabolic network. Spearman's rank correlation r = -0.74, *P* < 0.0001; Pearson's correlation r = -0.67, *P* < 0.0001. The centrality of an enzyme is equal to the mean length of network distances from the enzyme to all other enzymes in the networks (pairs of enzymes not connected by any path in the network were excluded from the calculation).

We will first discuss how network structure - specifically, an enzyme's position in the network - influences enzyme evolution. Generally, enzymes in central parts of metabolism such as the tricarboxylic acid cycle will have more neighbors than enzymes in peripheral metabolic pathways (Figure 1). The correlation shown in Figure 1 arises from the fact that more connected enzymes have a direct access to many network nodes and consequently have shorter path lengths to other enzymes in the network. The evolutionary constraints on a metabolic enzyme can be estimated through the normalized ratio of non-synonymous to synonymous substitutions per nucleotide site (K_a_/K_s_) that occurred in the gene coding for the enzyme [13]. A small K_a_/K_s _ratio suggests higher evolutionary constraints on the enzyme, that is, a smaller fraction of accepted amino acid substitutions. In our analysis, we used the average ratio K_a_/K_s _of unambiguous orthologs in four sequenced *Saccharomyces *species: *S. cerevisiae*, *S. paradoxus*, *S. bayanus*, and *S. mikatae *[14]. The average K_a_/K_s _values used in the main analysis were taken from the study by Kellis *et al*. [14]. We also recalculated the average ratios using the maximum-likelihood method of Yang and Nielsen [15] and obtained qualitatively similar results.

**Figure 2:**
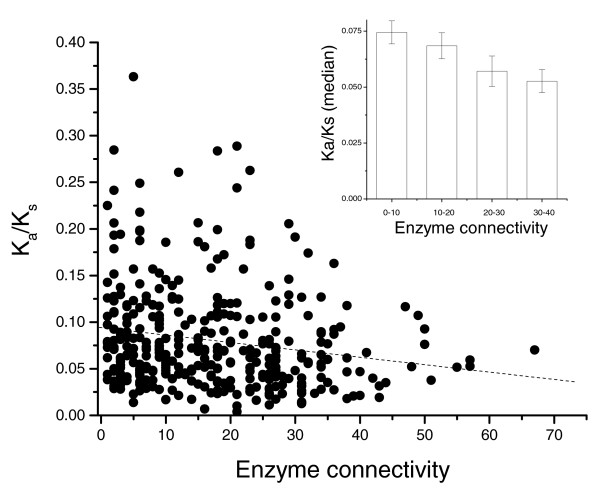
The relationship between enzyme connectivity in the yeast metabolic network and evolutionary constraint quantified by the K_a_/K_s _ratio. Spearman's rank correlation r = -0.20, *P* = 1.1 × 10^-4^; Pearson's correlation r = -0.18, *P* = 7 × 10^-4^. The connectivity of a metabolic enzyme is equal to the total number of other network enzymes producing or consuming the enzyme's reactants and products. K_a _is the fraction of amino acid replacement substitutions per amino acid replacement site on DNA; K_s _is the fraction of silent substitutions per silent site on DNA. The inset shows the histogram of binned enzyme connectivity versus median evolutionary constraint K_a_/K_s _(using the same data as in the main figure). The standard errors in each bin are also shown.

Figure 2 demonstrates a statistically significant negative correlation between the metabolic connectivity of an enzyme and the ratio K_a_/K_s _(Spearman's rank correlation r = -0.20, P = 1.1 × 10^-4^; Pearson's correlation r = -0.18, P = 7 × 10^-4^). The inset in Figure 2 shows that this negative association holds over a broad range of connectivities, and that it is not caused by a small number of highly connected proteins. Additional data file 2 demonstrates a weaker negative correlation between non-synonymous (amino acid changing) substitutions K_a _and gene connectivity (Spearman's rank correlation r = -0.13, P = 1.6 × 10^-2^). The reason is that using only K_a_, instead of the preferable K_a_/K_s_, as a measure of evolutionary constraints does not compensate for gene-specific differences in synonymous substitution rates and thus introduces additional noise in the data. Additional data file 3 shows that synonymous (silent) substitutions K_s _and enzyme connectivity are not significantly correlated (Spearman's rank correlation r = 0.056, P = 0.30). This is to be expected, as synonymous substitutions do not cause amino acid changes and are thus selectively neutral for the purpose of our analysis.

Why do highly connected enzymes show greater evolutionary constraint (smaller K_a_/K_s_)? One possibility is that this correlation is primarily mediated by the corresponding gene expression level [3]. Indeed, confirming previous observations [3], we found a significant negative correlation between the ratio K_a_/K_s _and mRNA expression levels (Spearman's rank correlation r = -0.33, *P* = 5.5 × 10^-10^; Pearson's correlation r = -0.30, *P* = 3.6 × 10^-8^). Information on mRNA expression of metabolic genes was obtained from the study by Holstege *et al*. [16] in which the number of mRNA molecules per cell was estimated based on microarray data. We also found a relatively weak correlation between connectivity and expression levels (Spearman's rank correlation r = 0.11, *P* = 4.6 × 10^-2^). Nevertheless, a partial correlation analysis - controlling for mRNA expression levels - between gene connectivity and evolutionary constraint K_a_/K_s _shows that enzymes in highly connected parts of the network evolve slowly independent of expression levels (Spearman's partial correlation r = -0.18, *P* = 1.4 × 10^-3^; the *P* value for Spearman's partial correlation was estimated by randomization).

### Enzymes that carry large metabolic fluxes evolve slowly

How well a metabolic network supports cell growth can be computationally quantified through the apparatus of metabolic flux analysis [17]. In flux balance analysis, the constraints imposed by stoichiometry and reversibility of chemical reactions are used to restrict the space of feasible metabolic fluxes. The constrained system can be subjected to an optimization procedure to obtain a flux distribution that maximizes some desirable metabolic property. Because cellular growth-rate is an important component of the fitness in a single-cell organism, biomass production is often used as the property being optimized. The predictions of flux balance analysis are often in good agreement with experimental results for *E. coli *[18,19] and *S. cerevisiae *[20].

To relate metabolic flux and the rate of enzyme evolution, we used flux balance analysis to calculate metabolic fluxes in the yeast metabolic network [10], maximizing growth on several different carbon sources (Table 1). Specifically, we asked whether flux through enzymatic reactions is associated with the evolutionary constraint K_a_/K_s _on the corresponding enzyme-coding genes. In this analysis, for enzymes catalyzing different chemical reactions, we used the reaction with the largest flux; if an enzyme had several isoenzymes (enzymes catalyzing the same reaction), we used the isoenzyme with the smallest ratio K_a_/K_s_. The growth conditions we used vary in the available carbon sources and in different uptake rates for oxygen (Table 1). The calculated distribution of flux values in the metabolic network is highly non-uniform [11] with several fluxes - usually representing glycolytic enzymes - more than two orders of magnitude larger than the rest (Additional data file 4). To eliminate the disproportionate effect of these large fluxes, we removed the fluxes that are two orders of magnitude larger than the median metabolic flux in the network (similar results are obtained with all fluxes). Figure 2 demonstrates, for an aerobic growth on glucose, a significant negative correlation between flux through individual enzymatic reactions and the ratio K_a_/K_s _(Spearman's rank correlation r = -0.31; *P* = 1.7 × 10^-3^; Pearson's correlation r = -0.24, *P* = 1.7 × 10^-2^). The correlation is clearly non-linear, and has an exponential shape. The results summarized in Table 1 show that similar associations exist between flux and evolutionary constraints K_a_/K_s _in other growth conditions on glucose and fructose - two natural carbon sources for yeast. Interestingly, the correlations between evolutionary constraint K_a_/K_s _and flux are substantially lower, and statistically insignificant, for acetate, a carbon source that may not dominate the natural yeast environment [21]. As we do not find any correlation between flux magnitude and connectivity (results not shown), the evolutionary constraints due to high fluxes are complementary to the connectivity constraints described above (Figure 2).

### Gene duplication correlation with connectivity and flux

**Figure 3:**
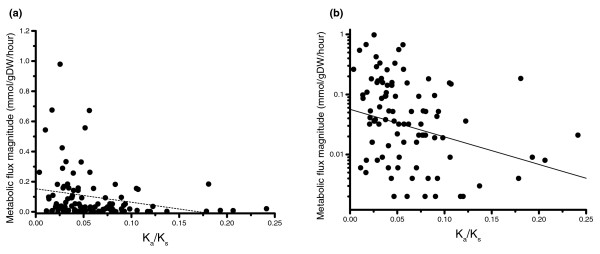
The relationship between metabolic flux and evolutionary constraint.**(a) **The relationship between metabolic flux values and evolutionary constraint K_a_/K_s _for aerobic growth on glucose. (maximal uptake rate for glucose 15.3 mmol/g dry weight (DW)/h; maximal oxygen uptake 0.2 mmol/gDW/h). Spearman's rank correlation r = -0.30; *P* = 2.7 × 10^-3^; Pearson's correlation r = -0.24, *P* = 1.7 × 10^-2^. The metabolic fluxes were calculated using flux balance analysis to maximize the cell growth rate. Fluxes more than two orders of magnitude larger than the median non-zero flux - representing large glycolytic fluxes - were excluded from the analysis. **(b) **The same as (a) but using log coordinates for the metabolic flux magnitude.

**Figure 4:**
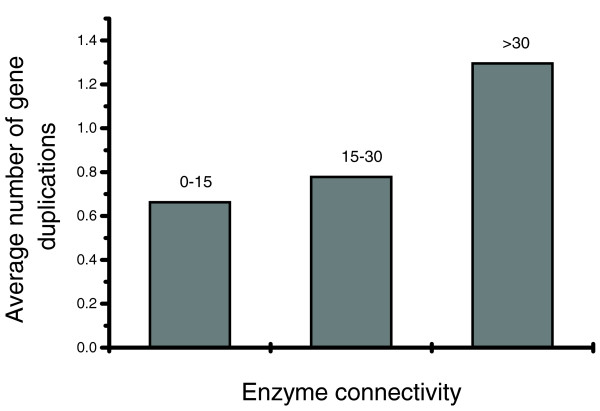
The relationship between enzyme connectivity and the average number of duplications in corresponding enzyme-coding genes. Enzymes with sequence identity larger than 40% over 100 or more aligned amino acids were considered as duplicates.

**Figure 5:**
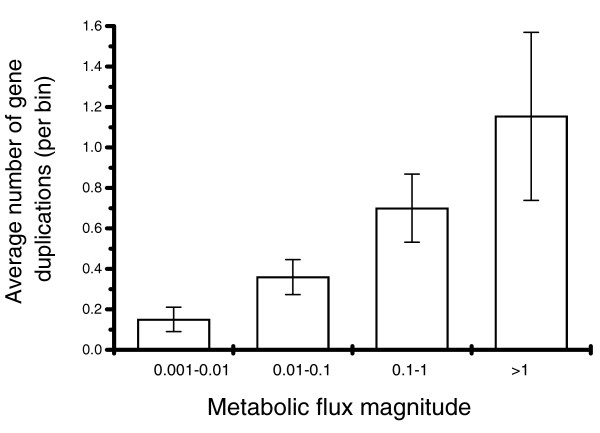
The relationship between the number of duplicates of an enzyme-coding gene and the magnitude of the metabolic flux through the enzymatic reaction. The results are shown for aerobic growth on glucose (maximal uptake rate for glucose 15.3 mmol/gDW/h; oxygen 0.2 mmol/gDW/h). Putative duplicate pairs with less than 40% amino acid similarity or less than 100 aligned amino acid residues were excluded.

Gene duplications have effects opposite from those of most amino acid changes: they may increase rather than reduce flux through an enzymatic reaction. We established that highly connected enzymes and enzymes with high associated flux are especially sensitive to amino acid changes (Figures 2 and 3). Are their enzyme-coding genes, conversely, also more likely to undergo duplication? Figure 4 shows that this is indeed the case for enzyme connectivity. The figure demonstrates an association between an enzyme-coding gene's number of duplicates and enzyme connectivity (only enzymes with sequence identity higher than 40% were considered as duplicates). Mean connectivity for genes with no duplicates is 15.0, and for genes with duplicates it is 19.2 (non-parametric Wilcoxon test, *P* = 1.4 × 10^-4^). This result suggests that duplicates of enzymes producing or consuming widely used metabolites are more likely to be retained in evolution. Figure 5 and Additional data file 5 demonstrate that a similar association exists between non-zero enzymatic flux through a reaction and the number of duplicates of the respective enzyme's coding gene. Specifically, the higher the flux through a reaction, the more duplicates an enzyme-coding gene has. Qualitative association between enzymatic flux and gene duplication was also recently shown by Papp *et al*. [22].

### Connectivity, essentiality, and metabolic robustness

Evolutionary constraints on enzymes are indirect indicators of metabolic robustness to amino acid changes, changes that a metabolic network tolerated for well over millions of years of evolution. Another type of biological robustness is that against complete gene deletions. Robustness against gene deletions can be derived from laboratory studies in which the effects of gene deletions on growth rate and other indicators of fitness are studied [23,24]. These studies determine essential genes, that is, genes whose elimination in one or more laboratory environments is effectively lethal. Our use of available essentiality data is motivated by the observation that highly connected proteins in protein interaction networks may be more likely to be essential to a cell [1]. We carried out analyses using data on essential genes derived from a large scale gene deletion study by Giaever *et al*. [23], and used the *Saccharomyces *genome database (SGD) [25] to collect the essentiality data.

**Figure 6:**
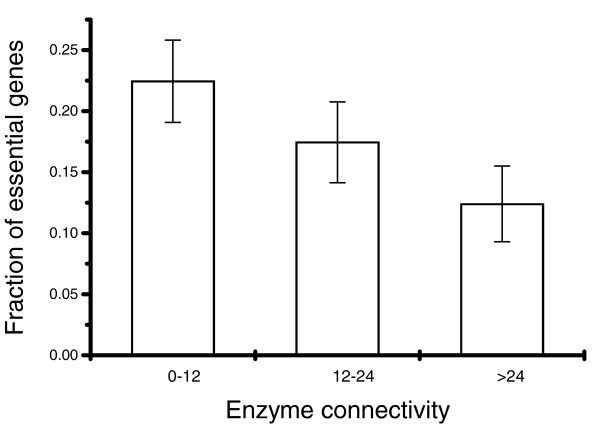
The relationship between enzyme connectivity and gene essentiality. The connectivity of a metabolic enzyme is equal to the total number of other network enzymes producing or consuming the enzyme's reactants and products. The information on gene essentiality was obtained from the systematic gene deletion study by Giaever *et al*. [23] using the SGD database [25].

Our analyses of essential enzyme-coding genes show the clearest deviations from behavior observed in protein interaction networks. In the metabolic network from which the most highly connected metabolites (such as ATP or hydrogen) have been excluded, the mean connectivity of essential enzymes is significantly smaller than the mean connectivity of non-essential enzymes (Figure 6). For the yeast network, mean connectivity of essential enzymes is 13.2, and for non-essential enzymes 17.5 (non-parametric Wilcoxon test, *P* = 4.0 × 10^-4^). No statistically significant difference in connectivity of essential and non-essential enzymes is observed if all metabolites are used to establish network connections. Consequently, highly connected metabolic enzymes are no more likely to be essential than low connected enzymes. Similarly, as Mahadevan *et al*. [26] demonstrated recently, removal of highly connected metabolites is no more essential than removal of low connected metabolites. As we show above, highly connected enzymes and enzymes carrying high fluxes are more likely to have duplicates (often with the same or similar biochemical function). This suggests that highly connected enzymes are no more likely to be essential because they often have duplicates that can compensate for loss-of-function mutations [27]. Indeed, we find that the average number of duplicates for essential metabolic enzymes is 0.19 while the average number of duplicates for non-essential enzymes is 0.8 (non-parametric Wilcoxon test, *P* = 8 × 10^-49^). In addition to gene duplications, flux rerouting may provide another mechanism to make highly connected genes less essential. In highly connected parts of a metabolic network, metabolic fluxes may be rerouted through alternative pathways after a loss-of-function mutation [19,28,29]. This does not hold for linear metabolic pathways at a metabolic network's periphery, where a loss-of-function mutation may be fatal because no rerouting is possible.

## Discussion

In sum, we demonstrate that both highly connected enzymes and enzymes that carry high metabolic fluxes in the yeast metabolic network have tolerated fewer amino acid substitutions in their evolutionary history. Why are enzymes carrying larger fluxes more constrained? The likely answer comes from the observation that most mutations affecting enzymatic activity may reduce rather than increase flux. Enzymes carrying high fluxes tend to have reaction products that enter a large number of metabolic pathways. Consequently, a mutational reduction in the activity of such enzymes should be more detrimental than a reduction in the activity of enzymes with lower flux.

We also show that the genes encoding enzymes with high flux have more duplicates. Importantly, we do not argue that duplications arise more frequently for genes whose products carry high flux, but that such duplications are more likely to be preserved in evolution, because of the advantage - higher flux - they provide. While a gene's duplicates can initially be preserved through an advantageous increase in metabolic flux, after divergence they may provide other functional benefits [30]. Divergence of metabolic genes in their expression and regulation is well-established for gene in intensely studied parts of metabolism, such as tricarboxylic acid cycle enzymes [31].

We found that the association between predicted enzymatic flux and evolutionary rate is most pronounced for carbon sources that dominate the natural environment of yeast. This suggests that one can use the association between flux and evolutionary constraint to search for conditions that dominated the evolution of metabolic networks. Similar analyses, which use genomic data to infer the environment that has shaped an organism's evolution, have been used before to show that carbon limitation may have influenced the evolution of the *E. coli *metabolic network more strongly than nitrogen limitation [19], and to show that yeast evolution favored fermentation over respiration [32].

A previous study by Hahn *et al*. [6] reported that, based on amino acid divergence, in the *E. coli *metabolic network there exists no statistically significant association between enzyme connectivity and evolutionary constraint. We emphasize that any contradiction between this earlier work and our results is only apparent. First, the earlier study was based on a much smaller set of enzymes (*n *= 108 as opposed to *n *= 350 here), and thus had less statistical power. Nevertheless, two different statistical measures in the previous study showed, like we do here, a negative association between connectivity and evolutionary constraint, albeit not at *P* < 0.05. Second, because of the lack of sufficient sequence information for a closely related sister species of *E. coli*, the previous study used only amino acid divergence K_*a *_and not the preferable K_*a*_/K_s _to indicate evolutionary constraint. In fact, the correlation between connectivity and K_a _is very similar between the present study and the previous work (Spearman's rank correlation r = -0.13, *P* = 1.2 × 10^-2 ^here versus Spearman's rank correlation r = -0.15, *P* = 7 × 10^-2 ^in the study by Hahn *et al*.).

It should not be surprising that the observed associations are weak in magnitude. The reason for the low magnitude is that many other factors influence the evolution of enzyme-coding genes. Two of these factors are gene expression levels (discussed in the paper) and constraints stemming from the tertiary and quaternary structure of enzymes, which may differ among enzymes (little is known about such constraints). The key point is that besides all these other factors, metabolic network function and structure also has a clear influence on protein evolution.

How do our results on the yeast metabolic network relate to earlier work on protein interaction networks? There, a similar relationship between protein connectivity and evolutionary constraint has been suggested [4,5]; however, this association exists for different reasons. Highly connected proteins in protein interaction networks may evolve slowly because a larger fraction of a highly connected protein's sequence is involved in protein interactions and may thus be evolutionarily constrained [4]. In contrast, high protein connectivity in the metabolic network is established not through protein-protein interactions, but through consumption or production of widely used metabolites. In metabolic networks, mutations in enzyme-coding genes - changing reaction rates and concentrations - may have especially deleterious consequences for widely used metabolites. Consequently, highly connected metabolic enzymes may evolve slowly due to functional as opposed to structural constraints. Our ability to consider fluxes through enzymes in a metabolic network allows us to relate the functional role of each enzyme in a network to its rate of evolution. Such a functional analysis of a genome-scale network has no counterpart in any other genome-scale network studied thus far.

In conclusion, our analysis of evolutionary constraints, gene duplication, and essentiality demonstrates that the structure and function of a metabolic network shapes the evolution of its enzymes. In the long run, system analyses of biological networks will allow us to increasingly place the evolution of genes in the larger context in which they operate, as building blocks of cellular networks.

## Materials and methods

### Metabolic network

We used a comprehensive collection of the yeast *S. cerevisiae *metabolic reactions by Foster *et al*. [10] to calculate metabolic enzyme connectivities. In addition to enzymatic reactions assigned to 671 open reading frames (ORFs), the collection contains reactions unassigned to known ORFs, transport reactions, and reactions represented by large macromolecular complexes. These reactions were used to calculate other enzyme connectivities but were excluded from the main analysis. Large macromolecular complexes (containing several ORFs) were represented by single enzymatic nodes in the calculation of connectivities for other metabolic enzymes. In order to include only functional relationships in the calculation of the enzyme connectivities, we excluded the 14 highly connected metabolites and co-factors (as described in the main text). As a result of the exclusion, a small fraction (5%) of network enzymes became disconnected from the network (they have zero connectivity). These enzymes were not included in the analysis.

### Flux balance analysis

Flux balance analysis (FBA) was used to obtain metabolic flux distribution as described previously [10,17,19]. The network by Forster *et al*. [10] was used in all flux balance calculations. The *in silico *network of yeast metabolism includes central carbon metabolism, transmembrane transport reactions, pathways responsible for the synthesis and degradation of amino acids, nucleic acids, vitamins, cofactors, and lipids. In total, the network consists of 733 metabolites and 1,175 metabolic reactions. In the flux-balance analysis, the constraints limiting nutrient uptake, reaction irreversibility, and steady-state conservation of metabolite concentrations are applied. The fluxes optimal for growth are then obtained by maximization of biomass production using linear optimization. Linear optimization was performed using the GNU Linear Programming Kit [33].

### Molecular evolution

We identified duplicates in the *S. cerecisae *genome using a previously described whole-genome analysis tool [34]. Briefly, the tool locates gene duplicates in a genome using BLASTP [35] and aligns them globally with the Needleman and Wunsch dynamic programming alignment algorithm [36]. Putative duplicate pairs with less than 40% amino acid similarity or less than 100 aligned amino acid residues were excluded; for the remaining pairs we calculated the number of substitutions per synonymous site (K_s_) and the number of substitutions per non-synonymous site (K_a_) using the maximum likelyhood models of Muse and Gaut [37] and Goldman and Yang [38].

The average K_a_/K_s_, K_a_, and K_s _values used in the analysis were obtained from the study by Kellis *et al*. [14]. In a complementary approach, we also recalculated the average ratios using the maximum-likelihood method of Yang and Nielsen [15] and obtained qualitatively similar results.

## Additional data files

The following additional data are available with the online version of this paper. Additional data file 1 is a figure showing examples of metabolic connectivity. (a) An example of the metabolic reaction network from sphingoglycolipid metabolism; metabolites are drawn as small circles (DHSP, sphinganine 1-phosphate; PETHM, ethanolamine phosphate; SPH, sphinganine; CDPETN, CDPethanolamine; ETHM, ethanolamine) and enzyme-encoding genes are shown in rectangles. (b) Metabolic connectivity of the *dpl1 *gene (solid edges), as defined by the reactions shown in (a). The *dpl1 *gene has a total of six metabolic connections: two established through ethanolamine phosphate (red edges); and four through sphinganine 1-phosphate (blue edges). Metabolic connections between other enzymes are show by dashed edges. Additional data file 2 demonstrates the relationship between enzyme connectivity and the average amino acid divergence K_a_. Spearman's rank correlation r = -0.13, P = 1.6 × 10^-2^. Additional data file 3 shows the relationship between enzyme connectivity and the average silent divergence K_s_. Spearman's rank correlation r = -0.056, P = 0.30. Additional data file 4 is a histogram of the calculated metabolic fluxes in the yeast network for aerobic growth on glucose (maximal uptake rate for glucose 15.3 mmol/g dry weight/h; oxygen 0.2 mmol/g dry weight/h). Note the small number of fluxes - representing glycolysis - with disproportionately large magnitudes. Similar flux distributions were also obtained for other growth conditions. Additional data file 5 shows the correlation between non-zero enzymatic flux through a reaction and the number of duplicates of the respective enzyme's coding gene. Additional data file 6 provides connectivity and evolutionary parameters (K_a_/K_s_, K_a_, K_s_) for yeast metabolic enzymes.

## Supplementary Material

Additional data file 1 (a) An example of the metabolic reaction network from sphingoglycolipid metabolism; metabolites are drawn as small circles (DHSP, sphinganine 1-phosphate; PETHM, ethanolamine phosphate; SPH, sphinganine; CDPETN, CDPethanolamine; ETHM, ethanolamine) and enzyme-encoding genes are shown in rectangles. (b) Metabolic connectivity of the *dpl1 *gene (solid edges), as defined by the reactions shown in (a). The *dpl1 *gene has a total of six metabolic connections: two established through ethanolamine phosphate (red edges); and four through sphinganine 1-phosphate (blue edges). Metabolic connections between other enzymes are show by dashed edges. Click here for file

Additional data file 2 The relationship between enzyme connectivity and the average amino acid divergence K_a_. Spearman's rank correlation r = -0.13, *P* = 1.6 × 10^-2^ Click here for file

Additional data file 3 The relationship between enzyme connectivity and the average silent divergence K_s_. Spearman's rank correlation r = -0.056, *P* = 0.30. Click here for file

Additional data file 4 Maximal uptake rate for glucose 15.3 mmol/g dry weight/h and for oxygen 0.2 mmol/g dry weight/h. Note the small number of fluxes - representing glycolysis - with disproportionately large magnitudes. Similar flux distributions were also obtained for other growth conditions. Click here for file

Additional data file 5 The correlation between non-zero enzymatic flux through a reaction and the number of duplicates of the respective enzyme's coding gene. Click here for file

Additional data file 6 Connectivity and evolutionary parameters (K_a_/K_s_, K_a_, K_s_) for yeast metabolic enzymes. Click here for file

## Figures and Tables

**Table 1:** Correlation between enzymatic flux magnitude and evolutionary constraint K_a_/K_s_

Uptake	Maximum uptake rates (mmol/gDW/h)	Spearman's rank correlation (*P *value) with zero fluxes	Spearman's rank correlation (*P *value) without zero fluxes
Glucose/oxygen	15.3/2.4	-0.28 (*P *= 3.8 × 10^-3^)	-0.25 (*P *= 3.6 × 10^-6^)

Glucose/oxygen	15.3/0.2	-0.31 (*P *= 1.7 × 10^-3^)	-0.22 (*P *= 5.7 × 10^-5^)
Glucose/oxygen	15.3/0.01	-0.26 (*P *= 9.3 × 10^-3^)	-0.21 (*P *= 1.2 × 10^-4^)
Fructose/oxygen	15.3/6.0	-0.27 (*P *= 6.4 × 10^-3^)	-0.20 (*P *= 2.5 × 10^-4^)
Fructose/oxygen	15.3/0.2	-0.25 (*P *= 1.3 × 10^-2^)	-0.20 (*P *= 1.8 × 10^-6^)
Acetate/oxygen	10.0/2.4	-0.08 (*P *= 0.45)	-0.21 (*P *= 9.2 × 10^-5^)
Acetate/oxygen	5.0/5.0	-0.010 (*P *= 0.39)	-0.19 (*P *= 3.7 × 10^-4^)

The correlation between enzymatic flux magnitude and evolutionary constraint K_a_/K_s _was calculated with and without enzymes carrying zero fluxes. gDW, grams dry weight.
