# Supplementary figures and images for: Influence of metabolic network structure and function on enzyme evolution
Source: Genome Biol. 2006 May 9;7(5):R39. doi: 10.1186/gb-2006-7-5-r39 (PMC1779518; doi:10.1186/gb-2006-7-5-r39)

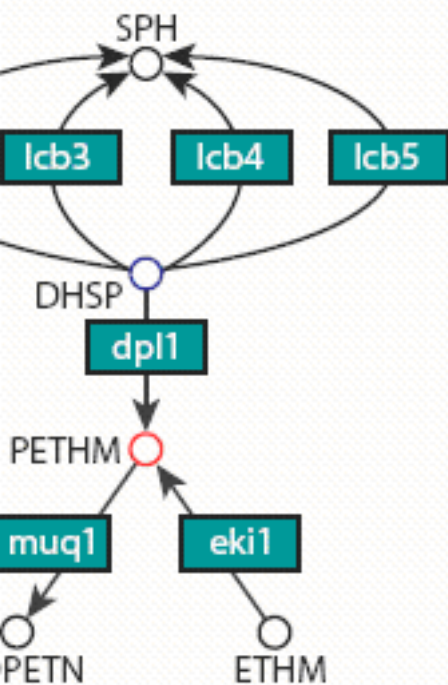

b.

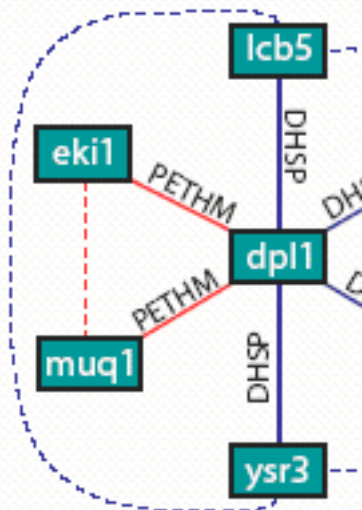

Supplement: Additional data file 1 — (a) An example of the metabolic reaction network from sphingoglycolipid metabolism; metabolites are drawn as small circles (DHSP, sphinganine 1-phosphate; PETHM, ethanolamine phosphate; SPH, sphinganine; CDPETN, CDPethanolamine; ETHM, ethanolamine) and enzyme-encoding genes are shown in rectangles. (b) Metabolic connectivity of the dpl1 gene (solid edges), as defined by the reactions shown in (a). The dpl1 gene has a total of six metabolic connections: two established through ethanolamine phosphate (red edges); and four through sphinganine 1-phosphate (blue edges). Metabolic connections between other enzymes are show by dashed edges. [file gb-2006-7-5-r39-S1.pdf]

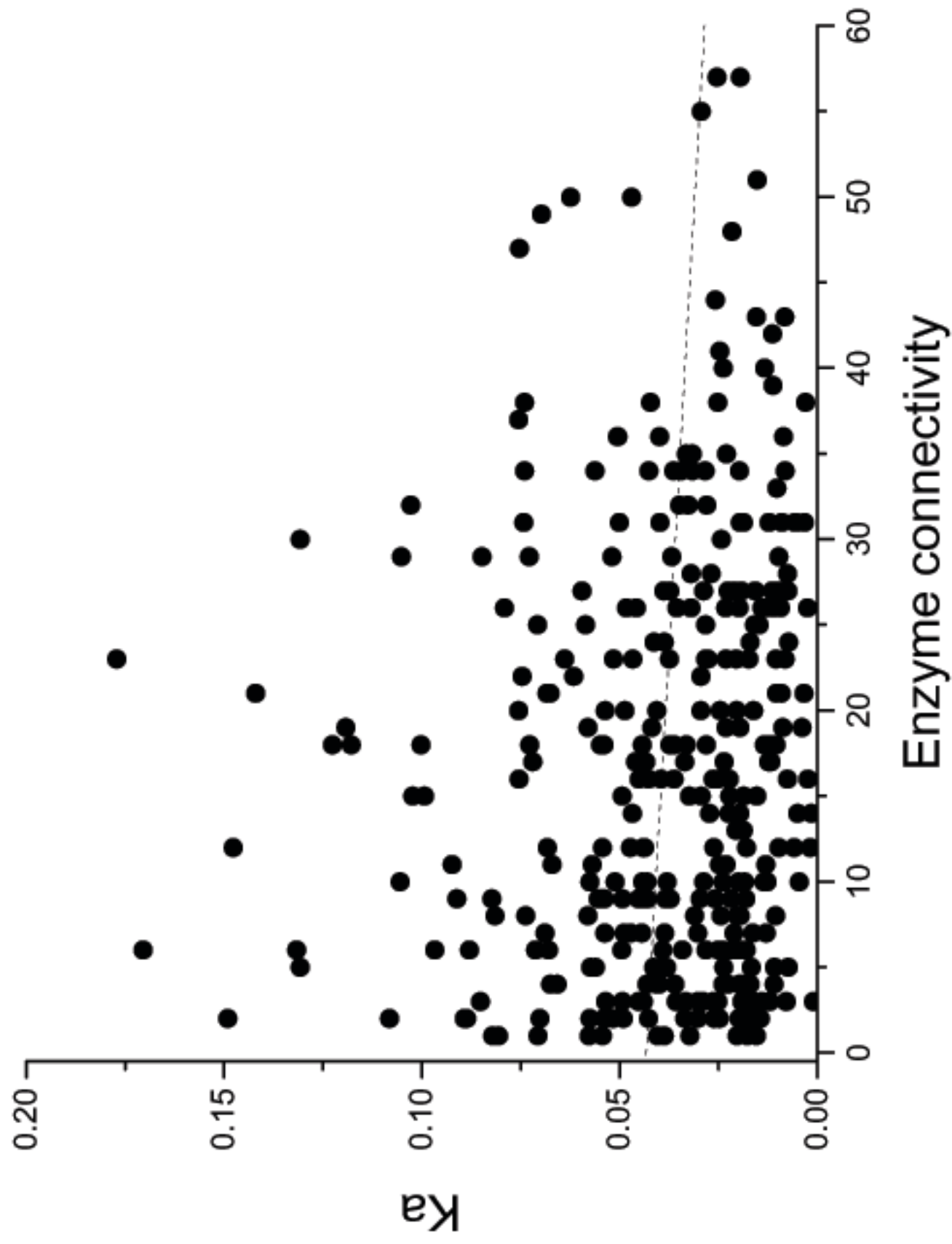

Supplement: Additional data file 2 — The relationship between enzyme connectivity and the average amino acid divergence Ka. Spearman's rank correlation r = -0.13, P = 1.6 × 10^-2^ [file gb-2006-7-5-r39-S2.pdf]

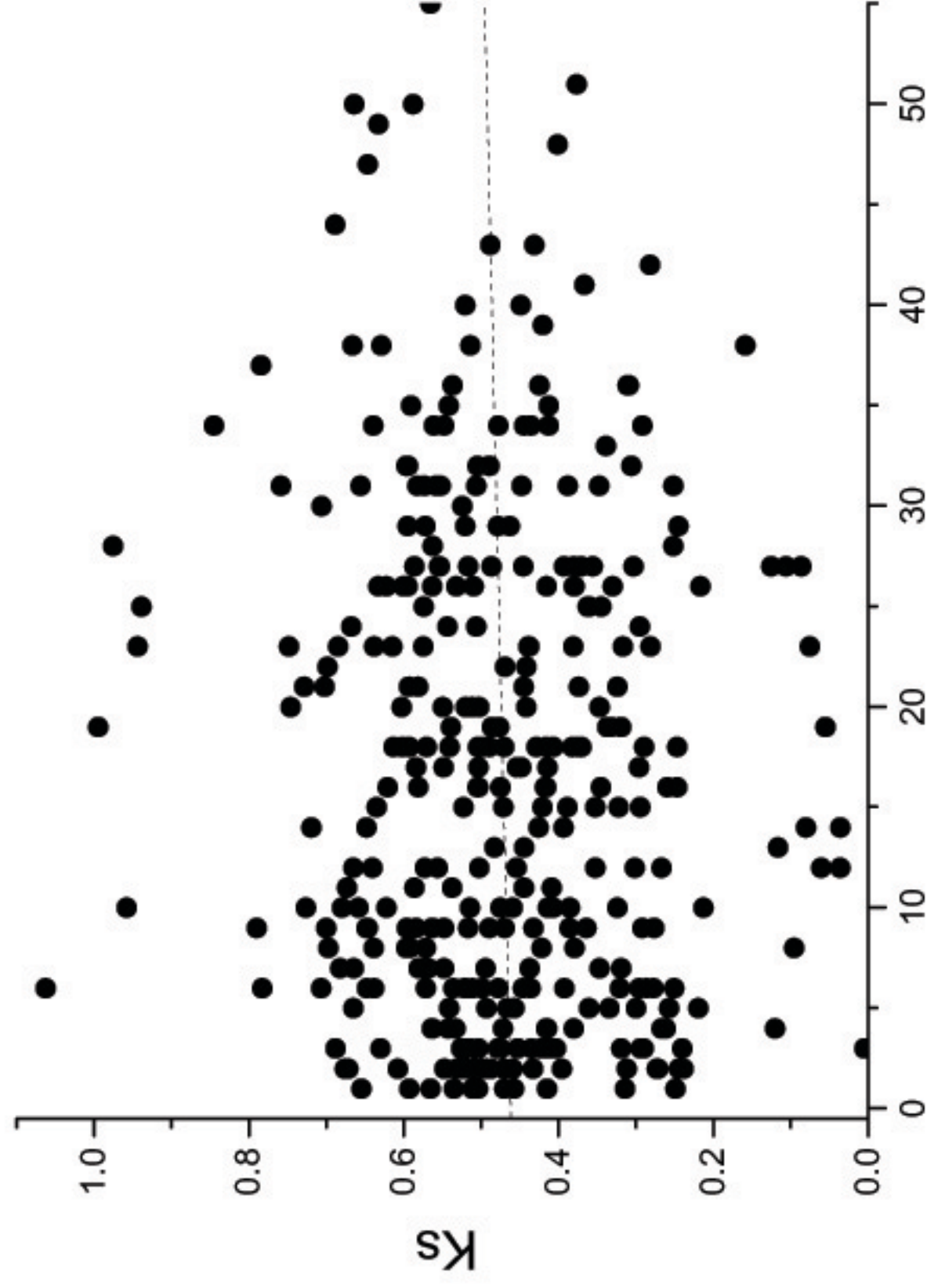

Enzyme connectivity

Supplement: Additional data file 3 — The relationship between enzyme connectivity and the average silent divergence Ks. Spearman's rank correlation r = -0.056, P = 0.30. [file gb-2006-7-5-r39-S3.pdf]

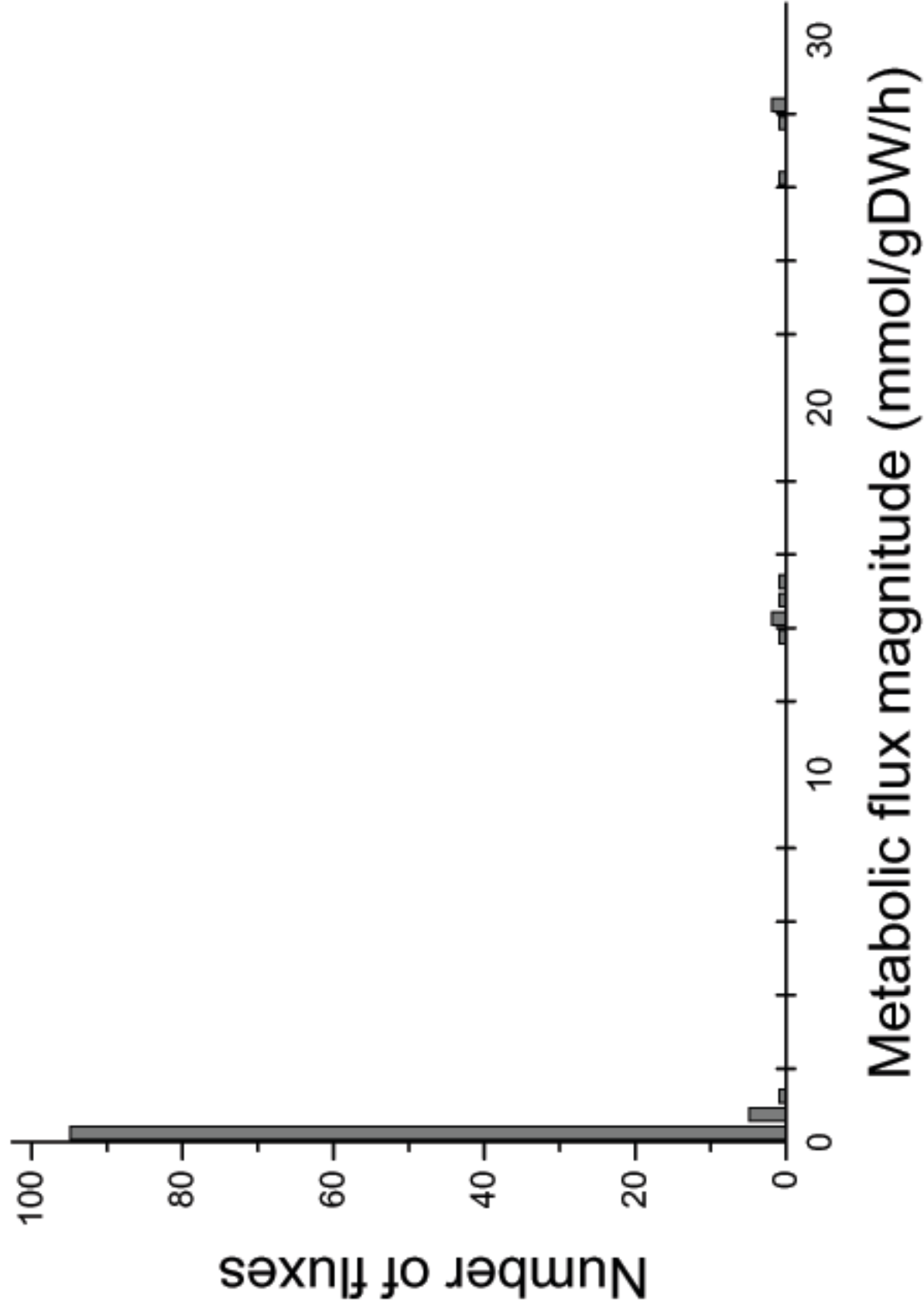

Supplement: Additional data file 4 — Maximal uptake rate for glucose 15.3 mmol/g dry weight/h and for oxygen 0.2 mmol/g dry weight/h. Note the small number of fluxes - representing glycolysis - with disproportionately large magnitudes. Similar flux distributions were also obtained for other growth conditions. [file gb-2006-7-5-r39-S4.pdf]
